# Draft genome of *Marinobacter salsuginis* SSi115 isolated from the brown seaweed *Sargassum ilicifolium* in Singapore

**DOI:** 10.1128/mra.00395-25

**Published:** 2025-08-11

**Authors:** Yen-Nhi H. Nguyen, Jessica A. Taylor, Su Xuan Gan, Prasha Maithani, Dalong Hu, Joao P. A. Pereyra, Rebecca J. Case

**Affiliations:** 1 Singapore Centre for Environmental Life Sciences Engineering (SCELSE), Nanyang Technological University (NTU) 54761, Singapore, Singapore; 2 Interdisciplinary Graduate Program, Nanyang Technological University (NTU) 54761, Singapore, Singapore; 3 School of Biological Sciences, Nanyang Technological University (NTU) 54761, Singapore, Singapore; 4 Singapore Centre for Environmental Life Sciences Engineering (SCELSE), National University of Singapore (NUS) 37580, Singapore, Singapore; 5 Saw Swee Hock School of Public Health, National University of Singapore (NUS) 37580, Singapore, Singapore; Montana State University, Bozeman, Montana, USA

**Keywords:** *Marinobacter*, *Marinobacter salsuginis*, *Sargassum ilicifolium*, brown seaweed, Singapore, type VI secretion system, hydrocarbonoclastic, bioremediation

## Abstract

*Marinobacter salsuginis* SSi115 was isolated from the brown macroalga *Sargassum ilicifolium*. Genome analysis revealed the presence of genes encoding a type VI secretion system involved in bacterial competition and host interactions. Genetic pathways for denitrification and hydrocarbon degradation are identified, indicating its potential role in bioremediation and biogeochemical cycling.

## ANNOUNCEMENT

The genus *Marinobacter*, belonging to the family *Phyllobacteriaceae* (class Alteromonadaceae), is found in diverse marine habitats and is a ubiquitous and abundant genera (1–16% relative abundance) when associated with the brown seaweed *Sargassum* (1). They chemotax to diatoms and dinoflagellates (2) and can antagonize algal hosts, including harmful algae (3, 4).

Thalli from three *Sargassum ilicifolium* (5) individuals were collected from the northern coast of St John’s Island, Singapore (1.222353 N 103.848702 E). Bacterial isolation was performed as described by Luk et al. (6). Briefly, the thallus was heat treated at 55°C, then stamped onto marine broth (Difco 2216) agar (1.5%) plates and incubated for 24 h at 30°C. Colonies were repeatedly subcultured to ensure purity. Biomass for genomic extraction was collected from a single colony inoculation of marine broth agar plates under aerobic conditions.

DNA was extracted using the Qiagen DNeasy Blood and Tissue Kit (Qiagen, Hilden, Germany) with the addition of RNase A. DNA library preparation used the TruSeq Nano DNA Library Prep Kit (Illumina, San Diego, USA). Sequencing was performed using an Illumina HiSeq X Ten platform, version 2.5, generating 150 bp paired-end reads. DNA library preparation and sequencing were performed by the SCELSE sequencing facility, NTU.

Quality filtering and adapter trimming were conducted using Trimmomatic, version 0.39 (7). Default parameters were applied for all software unless specified. Genome assembly was performed using the Shovill pipeline, version 1.1.0 (https://github.com/tseemann/shovill), using SPAdes, version 3.15.5 (8). The assembled genome was annotated using PGAP, version 6.7 (9). To identify plasmid sequences, the assemblies (Table 1) were analyzed with PlasmidFinder, version 2.0.1 (10), with no plasmids detected.

**TABLE 1:** Genomic features and accession numbers of *Marinobacter salsuginis* SSi115^*a*^

Isolate name	Genome size (bp)	Average coverage	No. of CDS^*b*^	No. of rRNAs	No. of tRNAs	G + C content (%)	No. of contigs	N50 (bp)	L50	Assembly accession no.	SRA accession no.	No. of reads	Average read length (bp)
SSi115^*a*^	4,347,050	33.7	3,962	6	49	57.3	18	3,207, 426	1	JBMIHS000000000	SRX28011657	7,979, 719	151

^*a*^ Singaporean *S. ilicifolium* isolate (SSi).

^*b*^ CDS, coding DNA sequence.

Taxonomic classification of the isolate genome was identified using the Genome Taxonomy Database Toolkit, version 1.7.0 (11), with the closest similarity to *M. salsuginis* 5N-3 (accession number BGZH00000000) (12) based on topology and average nucleotide identity (ANI). Phylogenetic relatedness between the isolate and the publicly available *M. salsuginis* reference genome was determined using the Orthologous Average Nucleotide Identity Tool, version 0.93.10 (13) and the Genome-to-Genome Distance Calculator, version 3.0 (14), to calculate the ANI and digital DNA–DNA hybridization (dDDH) scores, respectively. The isolate genome had >95% ANI and >70% dDDH scores against the reference genome, above the species threshold (15).

The *M. salsuginis* SSi115 genome encodes genes for core components of a type VI secretion system (T6SS), including *vgrG*, *hcp*, *tssA*, *tssH* (*clpV* homolog), *tssA*, *tssM*, *tssE*, *tssF*, *tssG*, *tssK*, and *tssJ* (16). The T6SS has been shown to be predictive of bacterial competition (17) or interaction with marine eukaryotic hosts (18), potentially contributing to the ecological fitness and host associations of *M. salsuginis* SSi115 in marine environments. The Kyoto Encyclopedia of Genes and Genomes (19) database identified genes (*narG*, *norB*, and *nosZ*) in the denitrification pathway and genes (*xylC*, *dmpK*) in the pathway to degrade monoaromatic hydrocarbons. These features suggest *M. salsuginis* SSi115 interacts with other marine organisms, can play a role in biogeochemical cycling, and could be a hydrocarbonoclastic bacterium involved in bioremediation processes (20).

## Data Availability

The SRA data were deposited in NCBI, and the complete genome sequence was deposited in GenBank under the accession numbers listed in Table 1.
